# Swin Transformer and the Unet Architecture to Correct Motion Artifacts in Magnetic Resonance Image Reconstruction

**DOI:** 10.1155/2024/8972980

**Published:** 2024-05-02

**Authors:** Md. Biddut Hossain, Rupali Kiran Shinde, Shariar Md Imtiaz, F. M. Fahmid Hossain, Seok-Hee Jeon, Ki-Chul Kwon, Nam Kim

**Affiliations:** ^1^Department of Information and Communication Engineering, Chungbuk National University, Cheongju-si 28644, Chungcheongbuk-do, Republic of Korea; ^2^Department of Electronics Engineering, Incheon National University, 119 Academy-ro, Yeonsu-gu, Incheon 22012, Republic of Korea

## Abstract

We present a deep learning-based method that corrects motion artifacts and thus accelerates data acquisition and reconstruction of magnetic resonance images. The novel model, the Motion Artifact Correction by Swin Network (MACS-Net), uses a Swin transformer layer as the fundamental block and the Unet architecture as the neural network backbone. We employ a hierarchical transformer with shifted windows to extract multiscale contextual features during encoding. A new dual upsampling technique is employed to enhance the spatial resolutions of feature maps in the Swin transformer-based decoder layer. A raw magnetic resonance imaging dataset is used for network training and testing; the data contain various motion artifacts with ground truth images of the same subjects. The results were compared to six state-of-the-art MRI image motion correction methods using two types of motions. When motions were brief (within 5 s), the method reduced the average normalized root mean square error (NRMSE) from 45.25% to 17.51%, increased the mean structural similarity index measure (SSIM) from 79.43% to 91.72%, and increased the peak signal-to-noise ratio (PSNR) from 18.24 to 26.57 dB. Similarly, when motions were extended from 5 to 10 s, our approach decreased the average NRMSE from 60.30% to 21.04%, improved the mean SSIM from 33.86% to 90.33%, and increased the PSNR from 15.64 to 24.99 dB. The anatomical structures of the corrected images and the motion-free brain data were similar.

## 1. Introduction

Magnetic resonance imaging (MRI) is used to diagnose many conditions, and there is no radiation exposure [1]. MRI image resolution and the signal-to-noise ratio have increased as the technology progressed, but longer scan times increase susceptibility to motion artifacts (MAs) [2]. Patient movement compromises the k-space [3]; blurring or ghosting artifacts appear in reconstructed images. Especially for patients who are unable to control their movements, such motion distortions may affect the diagnosis. Therefore, the need to deal with subject motion is always considered by MRI researchers. MRI data are collected in the Fourier domain, often termed the k-space. Every point in that space represents an image frequency; a change in even one k-space location affects the entire image. Both nonrigid and rigid motions are frequently visible on MRI [4]. Nonrigid motion refers to spontaneous deformation that may be physiological, such as cerebrospinal fluid (CSF) flow, respiration, coughing, and swallowing. Rigid motion is generated by spontaneous random gestures and is more common in “disobedient” subjects such as children or those with degenerative neurological disorders (e.g., Parkinson's disease). Motion parameters [4] are derived directly from raw data by reducing the errors associated with motion-related data inconsistencies. However, it is difficult to accurately specify the motion parameters given the nonconvex nature of the computation and the long processing time.

Several strategies that prevent motion or correct artifacts have been developed to solve such motion-related issues. The most basic method is inhibition of patient movement via breath-holding or anesthesia [5]. An attempt has been made to combine this with parallel imaging (PI) to reduce the burden on the patient [6]. This approach minimizes scan times by using a smaller number of frequencies. However, the scans remain susceptible to artifacts if subjects cannot control their movements. MRI navigators [7] are commonly employed to measure patient motion; these navigators have in-bore cameras and markers [8]. These prospective techniques yield partial k-spaces based on trajectory information [9]. Retrospective methods that process data after MRI [10] are aimed at correcting motion. Such retrospective techniques are used in conjunction with algorithms that estimate motion without initially gathering motion data. However, in these scenarios, complicated and unexpected patient motions restrict computation. In general, the traditional approaches are associated with additional costs, increased scan times [11], and sequence changes [12]. The resolution of MAs is of major clinical importance; it is essential to automate the correction of MRI motion abnormalities without the use of navigators.

**Figure 1:**
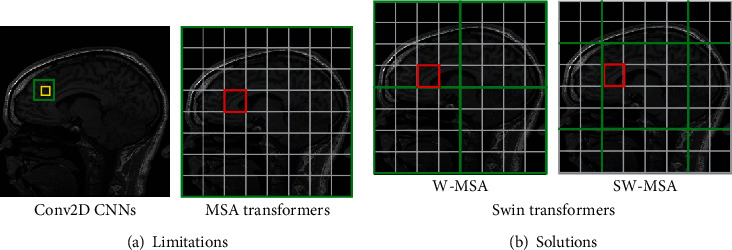
The receptive fields for two-dimensional convolution (Conv2D), multihead self-attention (MSA), and shifted window-based MSA (W-MSA/SW-MSA) are shown in (a) and (b). Receptive field of operation, green box; pixel, yellow box; self-attention patch, red box.

Recently, deep learning (DL) has proven extremely effective for image processing [13], including image reconstruction [14] and detection [15]. The complex and nonlinear properties of DL are combined with unsupervised or supervised learning. DL is now widely used to reconstruct MRI images [16], as well as for motion correction, because motion-tracking equipment, extended scan times, and sequence modification are not required [17]. A neural network (NN) is trained using large datasets that typically employ motion-contaminated images as inputs and motion-free images as labels. A specific type of NN, the convolutional NN (CNN), improves hidden attribute extraction because the hierarchical structure is extremely deep [18]. Convolutions efficiently extract features; CNNs have always dominated the field of computer vision (CV). Many attempts have been made to use DL paradigms to reduce MAs. Meding et al. [19] developed a data-driven, fully automated CNN to determine whether MAs in images impacted the quality of MRI recordings. Eschewing the interscan method, they employed a binary classification model to distinguish “motion” and “no motion.” The Inception-ResNet [20] architecture-based motion correction network (MoCo-Net) [11] is a two-dimensional (2D) encoder-decoder system trained by simulated motions. Automatic multistream CNNs were developed by Oksuz et al. [21] and Zhang et al. [22] to find MAs in cardiac MRI scans. Kustner et al. [23] used a variational autoencoder (VAE) and a generative adversarial network (GAN), i.e., image-to-image translation techniques, for retrospective correction of rigid and nonrigid MAs. The GAN yielded the best motion-free realistic images. Johnson and Drangova [24] created a three-dimensional (3D) version of a 2D pixel-to-pixel network based on a conditional GAN and used this for 3D rigid-body motion correction of brain MRI scans. Test volumes with motion-contaminated data were qualitatively explored. Duffy et al. reviewed 3D CNNs used for retrospective MA reduction and their capacity to recognize morphological modifications in brains with Parkinson's disease [25]. The network accelerated motion estimation and reduction (NAMER) [26] is a motion correction network that combines artifact recognition with motion estimation. Wang et al. [12] used a CNN that employed data fidelity to correct 2D motions. Even when used to correct 3D motions, the CNN outperformed a simple data fidelity model in terms of image quality. To eliminate MAs in MRI scans, Liu et al. [27] created a deep residual model that coupled distorted inputs to outputs with fewer artifacts. However, such correction methods cannot be used if patient motions are unpredictable; all of the studies cited above focused on fundamental (fixed) motion patterns [11]. Image reconstruction has long proven highly challenging. Appropriate network training requires novel data generation methods that handle MAs arising for various reasons. Also, all methods described above are CNN-based; the receptive field of a CNN is constrained by the network depth and the convolutional kernel. A large kernel significantly increases computing costs, and a deep network may be associated with a vanishing gradient. Convolution is locally sensitive and independent of distance and is thus the basis of CNN feature extraction (Figure 1(a)). Two-dimensional convolution (Conv2D) is not long-range-dependent and is therefore locally sensitive.

A recently developed unique transformer was initially used for natural language processing (NLP) [28], and later for medical image analysis [29], based on a self-attention (SA) mechanism [30]. However, it is not easy to switch from NLP to CV tasks. First, the scales differ. Unlike language components (such as the word tokens of NLP tasks), visual components (such as pixels) of CV tasks frequently vary in terms of scale. Second, resolution may be an issue. Pixels in images or frames are typically of significantly higher resolution than words in phrases. However, given the improved image reconstruction and synthetic ability of transformers acting on real images, transformers are now linked to MRI in a variety of ways. Newer transformers perform well on tasks requiring reconstruction [31] and superresolution [32]. The “shifted windows” (Swin) transformer [33] uses Swin-based multihead self-attention (MSA) to replace the typical MSA. Swin transformer-based networks perform extraordinarily well, even outperforming CNN-based techniques; Swin-based systems serve as cutting-edge solutions for complex vision-based tasks. Figures 1(a) and 1(b) show that limiting the scale of SA in a local window is a trade-off that reduces computational complexity. The receptive fields of MSA and windows-MSA/Swin-based-MSA (W-MSA/SW-MSA) are greater than those of Conv2D. W-MSA and SW-MSA are utilized by Swin transformers to shift windows; MSA operates over the entire image space. As the cross-window relationship may be disregarded if all attention actions proceed in fixed windows, W-MSA and SW-MSA alternate throughout the later transformer layers.

We present the Motion Artifact Correction by Swin Network (MACS-Net), a unique mode of MA correction based on a Swin transformer module and the Unet architecture [34] that ensures rapid MRI scan reconstruction. This integration of Swin transformer and Unet architecture leads to a model that generalizes well across a range of image sizes and complexities, as it can capture both the global structure and local details of the image.

The major contributions of this study are as follows:

- We integrated the Unet framework with a Swin transformer block (STB) to develop a novel network that identifies and corrects MAs during MRI scan reconstruction
- A dual upsample block is introduced including both subpixel and bilinear upsample algorithms. Experimentally, this outperformed initial upsampling alone
- This MACS-Net performs on the publicly available brain motion artifact datasets

## 2. Materials and Methods

The artifact-free ground truth image (*G*) is reconstructed from the fully sampled motion-free k-space (*k*) by executing the inverse Fourier transform (IFT):

(1) $$\begin{array}{c} G=\text{IFT}\left(k\right). \end{array}$$

During restoration, a reconstructed artifact image (*y*) is denoted as

(2) $$\begin{array}{c} Y=D\left(G\right)+m, \end{array}$$

where *D*(.) is the degradation function and *m* represents the MAs. DL mitigates such artifacts (*m*) by learning how they impact MRI scans. MA reduction was designed and simulated using Swin transformations and a Unet architecture; we employed a dataset with authentic MAs.

### 2.1. Dataset Generalization and Preprocessing

It is difficult to pair motion-contaminated and motion-free k-spaces; most state-of-the-art approaches are thus trained using synthetically corrupted images derived from motion-free data. A phase shift can be added to the frequency domain of a k-space to simulate MAs, or rotations and translations can be implemented in the spatial domain. Several degrees of motion severity can be introduced by varying the extent of motion. In our analysis, we used the Movement-related Artifacts (MR-ART) [35] dataset with information on the heads of 148 healthy adults (95 women and 53 men) who underwent 3D structural T1-weighted MRI. Both motion-free and motion-affected data from the same volunteers are available. T1-weighted 3D magnetization-prepared rapid gradient-echo (MP-RAGE) anatomical images were collected via two-fold in-plane generalized autocalibrating partial parallel acquisition (GRAPPA) acceleration with an isotropic spatial resolution of 1 mm^3^ (repetition time (TR) = 2,300 ms, echo time (TE) = 3 ms, inversion time (TI) = 900 ms, flip angle (FA) = 9°, and field of view (FOV) = 256 × 256 mm). No participant (age range: 18–75 years; median age: 25.16 years; interquartile range: 10.50 years) had any neurological or psychiatric condition. Images were captured with the heads motionless and next moving both slightly and markedly; there are three datasets for each participant. For each subject, three T1-weighted structural scans were obtained using the same parameters but with three different settings: STAND (no motion), M1 (brief motion, within 5 s), and M2 (longer motion, 5–10 s). A fixation point was set in the middle of the display during each acquisition, and participants were told to look at the point. Participants were advised to remain completely still during the STAND scan and to nod their heads during the M1 and M2 scans. Brain Imaging Data Structure (BIDS) software organized and anonymized the 3D images. The Pydeface tool [36] was used to erase facial details. The authors provided the basic image quality parameters of the MRI quality control (MRIQC) [37] reports on all scans; this rendered labelling more precise. To describe how MAs affected the clinical utility of the images, board-certified neuroradiologists awarded artifact scores to each scan. For each subject, the STAND scan was rated as good (score 1), the M1 scan as medium (score 2), and the M2 scan as poor (score 3).

Among the 148 volunteers' (95 women and 53 men) data files, 80 (50 women and 33 men) were used for training, 48 (30 women and 15 men) for validation, and 20 (15 women and 5 men) for testing. Each file contained three sets of data (ground truth, low motion, and moderate long motion). Each set contained 179 sagittal cross-sectional T1-weighted (256 × 256) MRI image slices. During the training of the networks, normalized high MA images served as inputs and motion-free images as outputs. On the other hand, the comparative networks were evaluated by both low and high MA images. No data augmentation was applied during training.

### 2.2. Network Architecture

**Figure 2:**
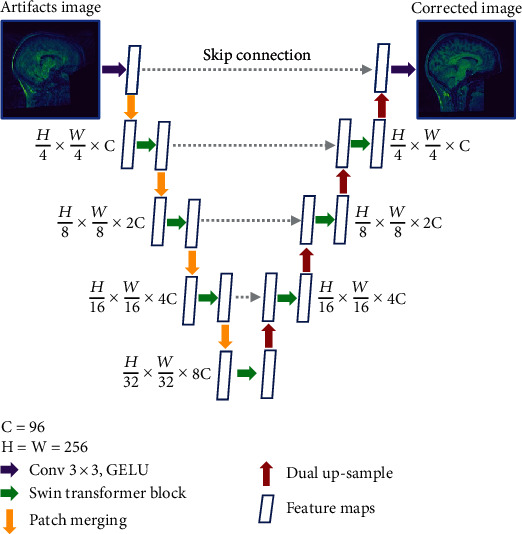
The Swin transformer-based method for correction of MAs. C: number of channels; GELU: Gaussian error linear unit.

The NN for MA correction using the Swin transformer (MACS-Net) is shown in Figure 2. In MACS-Net, the input image is divided into nonoverlapping patches of a fixed size, which is commonly referred to as the “patch size.” The patch size determines the granularity at which the model processes the image and affects the spatial resolution of the output features. A smaller patch size allows the model to capture more fine-grained details in the image, but it increases the computational cost and memory requirements. Conversely, a larger patch size reduces the computational cost and memory requirements, but it may result in the loss of fine-grained details. The patch size is determined by the window size and the number of patches that cover the entire image. The input image size of this proposed network is 256 × 256 pixels, and the window size is 32; therefore, the number of patches is (256/32) × (256/32) = 64, and the patch size is 4 pixels. There are three modules for shallow feature extraction (SFE), Unet feature extraction (UFE), and reconstruction.

#### 2.2.1. SFE

Artifacts input images *y* ∈ *R*^*h*×*w*×3^, where *h* and *w* are the height and width of each distorted image. We used a single 3 × 3 convolutional layer *M*_SFE_(.) to obtain low-frequency information (such as colors or textures) from the inputs. A representative shallow feature *F*_*s*_ ∈ *R*^*h*×*w*×*c*^ is derived as follows:

(3) $$\begin{array}{c} F_{s}=M_{\text{SFE}}\left(y\right), \end{array}$$

where *c* is the number of channels that analyze shallow features; here, *c* = 96.

#### 2.2.2. UFE

Subsequently, high-level multiscale deep features *F*_*d*_ ∈ *R*^*h*×*w*×*c*^ were obtained from each shallow feature *F*_*s*_ via UFE:

(4) $$\begin{array}{c} F_{d}=M_{\text{UFE}}\left(F_{s}\right), \end{array}$$

where *M*_UFE_(.) is the Unet framework of the STB with eight Swin transformer layers (STLs) in each STB. The STB and STL are described in detail below.

#### 2.2.3. Reconstruction Module (RM)

An artifact-free image $\hat{x}=R^{h\times w\times 3}$ is generated from a deep feature *F*_*d*_ via 3 × 3 convolution  *F*_RM_(.):

(5) $$\begin{array}{c} \hat{x\;}=M_{\text{RM}}\left(F_{d}\right), \end{array}$$

where $\hat{x}$ is the artifact-corrected output from the input artifact-containing image (*y*).

### 2.3. Resizing Module

As Unet feature maps vary in scale, resizing modules that engage in down- and upsampling is required. These MACS-Net modules employ a patch merging and a new dual upsampling technique, respectively.

#### 2.3.1. Patch Merging

To connect the input features of each cluster of adjacent 4 × 4 patches to the down-sampling module, we employed a hierarchical vision transformer [33] and a linear layer to produce the output features for the desired number of channels. Alternatively, input feature map unfolding could serve as the first stage of convolution.

#### 2.3.2. Dual Upsampling

**Figure 3:**
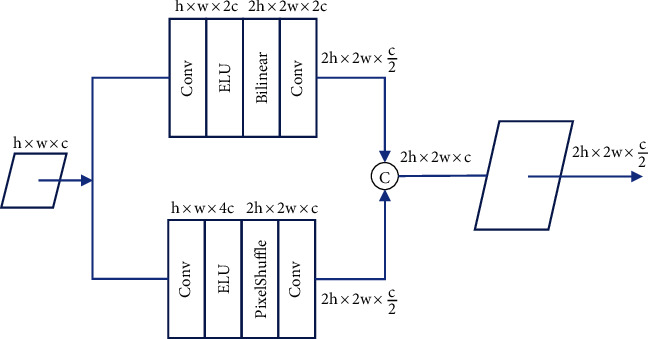
The new dual upsampling module employing both subpixel and bilinear approaches.

The basic Swin-Unet [38] uses a patch-expanding approach for upsampling, which is almost equivalent to transpose convolution but deals with block effects more rapidly. To avoid image distortion, we used a new dual upsampling method that combines the bilinear and PixelShuffle [39] techniques. Bilinear upsampling considers the nearest four pixels surrounding each pixel in the lower-resolution image. It then performs a weighted average of these pixel values to calculate the corresponding pixel value in the higher-resolution image. It is simple and fast but may not produce the best results, especially when the upsampling factor is large. PixelShuffle upsampling rearranges the elements of the tensor by grouping neighbouring elements and moving them to different spatial locations in the output tensor. It is more complex and computationally demanding but provides better results. The combination ensures high-quality upsampled images and exploits the computational efficiency of bilinear upsampling. Here, the exponential linear unit (ELU) served as the activation function.  Figure 3 shows the architecture of the new upsampling module.

### 2.4. Swin Transformer Block

Transformers [40] are at least as effective as CNNs for image classification [41] and NLP. However, two fundamental issues arise when using a transformer to handle a visual task. First, the scales of images and sequences differ markedly. A transformer requires a number that is similar to the sum of squares of all parameters in a one-dimensional (1D) sequence; a transformer cannot handle long sequences effectively. Second, transformers do not make dense predictions, such as segmentation, effectively, such that they must be completed at the pixel level [42]. The Swin transformer handles several pixel-wise vision tasks by using Swin to reduce the number of parameters.

**Figure 4:**
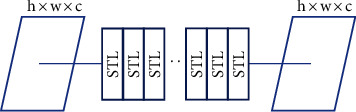
A Swin transformer block (STB) with eight Swin transformer layers (STLs).

Figure 4 shows that an STB replaces the conventional convolution layer of the Unet extraction module. The fundamental NLP transformer layer [30] serves as the STL foundation. Each STL number is a multiple of two. Window multihead self-attention (W-MSA) and shifted-window multihead self-attention (SW-MSA) are represented by separate STLs. Certain issues arise when transformers directly perform CV tasks, as indicated in Introduction. A cyclic shift technique was used to reduce computation time with maintenance of the properties of convolution, i.e., the rotation, translation, and size invariance of the relationship between the receptive field and the layers.

### 2.5. Swin Transformer Layer (STL)

**Figure 5:**
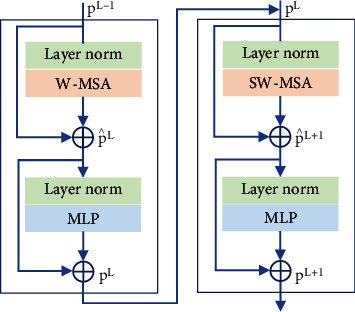
Two Swin transformer layers (STLs).

The STL uses Swin, unlike the traditional MSA module. Two sequential STLs are shown in Figure 5. The next two transformer layers use the W-MSA and the SW-MSA modules, respectively. In W-MSA, the input sequence is divided into multiple fixed-size segments or “windows,” and self-attention is applied to each window independently. This approach helps to reduce the computational cost of self-attention, as it allows for parallel processing of the windows. In SW-MSA, the windows are shifted by a certain amount (e.g., half the window size) to overlap with adjacent windows. This approach assists in alleviating the information loss issue while allowing for parallel processing.

The continuous STLs based on the window-partitioning approach can be written as

(6) $$\begin{array}{c} {\overset{\wedge }{p}}^{L}=\text{W}-\text{MSA}\left(\text{LN}\left(p^{L-1}\right)\right)+p^{L-1},\\ p^{L}=\text{MLP}\left(\left(\text{LN}\left({\overset{\wedge }{p}}^{L}\right)\right)\right)+{\overset{\wedge }{p}}^{L},\\ {\overset{\wedge }{p}}^{L+1}=\text{SW}‐\text{MSA}\left(\text{LN}\left(p^{L}\right)\right)+p^{L},\\ p^{L+1}=\text{MLP}\left(\text{LN}\left({\overset{\wedge }{p}}^{L+1}\right)\right)+{\overset{\wedge }{p}}^{L+1}, \end{array}$$

where LN(:) indicates layer normalization and MLP is a multilayer perceptron with two completely linked layers activated by GELUs. ${\overset{\wedge }{p}}^{L}$ and *p*^*L*^ are the outputs of the *L*^th^ block of (S)W-MSA and the MLP module, respectively.

## 3. Assessment Metrics and Experimental Setup

### 3.1. Assessment Metrics

The normalized root mean square error (NRMSE), structural similarity index measure (SSIM), and peak signal-to-noise ratio (PSNR) were used to evaluate network performance. The NRMSE analyzes pixel differences between a reference and network predictions:

(7) $$\begin{array}{c} \text{NRMSE}\left(G,X\right)=\frac{\sqrt{\left(1/N\right)\sum_{i=1}^{N}{\left(G_{i}–X_{i}\right)}^{2}}}{\max\left(G\right)–\min\left(X\right)}, \end{array}$$

where *N* is the image size, *i* is the slice position, and *G* and *X* are the motion-free (ground truth) and reconstructed (output) images, respectively. The SSIM perceptual index compares the mutual dependencies of adjacent pixels in terms of structural traits, contrast, and brightness, i.e., determines how similar the two images are. To compute the SSIM between *G* and *X*, we employ

(8) $$\begin{array}{c} \text{SSIM}\left(G,X\right)=\frac{\left(2\mu_{G}\mu_{X}+q_{1}\right)\left(2\sigma_{GX}+q_{1}\right)}{\left(\mu_{G}^{2}+\mu_{X}^{2}+q_{1}\right)\left(\sigma_{G}^{2}+\sigma_{X}^{2}+q_{1}\right)}, \end{array}$$

where *μ*_*G*_ and *μ*_X_ are the average values of *G* and *X*, respectively, *σ*_*G*_^2^ and *σ*_*X*_^2^ are the respective pixel variances, and *σ*_*GX*_ is the covariance. *q*_1_ and *q*_2_ are used for data division:

(9) $$\begin{array}{c} q_{1}={\left(0.01v\right)}^{2},\\ q_{2}={\left(0.03v\right)}^{2}, \end{array}$$

where *v* = max(*G*)–min(*X*).

The PSNR parameter quantifies the connection between the peak potential signal power and noise that reduces signal fidelity:

(10) $$\begin{array}{c} \text{PSNR}\left(G,X\right)=10\log_{10}\left(\frac{255}{\sqrt{\left(1/N\right)\sum_{i=1}^{N}{\left(G_{i}–X_{i}\right)}^{2}}}\right). \end{array}$$

These three metrics are frequently used to evaluate image reconstruction. Higher SSIM and PSNR values, but lower NRMSE values, indicate better images.

### 3.2. Experimental Setup

For model training and testing, we used a Windows 10 Pro-64-bit computer with an Intel Core i7-9800X 3.80 GHz processor, 128 GB of RAM, and an NVIDIA GeForce RTX 2080Ti graphics processing unit. All models were implemented using the PyTorch v1.11.0 modules of PyCharm and Python 3.8. Commencing at 10^−4^, the learning rate decreased every 20 epochs with a decay factor of 0.96. During training, 500 epochs were completed with a small batch size of 8. All tested networks were implemented identically.

### 3.3. Loss Function

**Figure 6:**
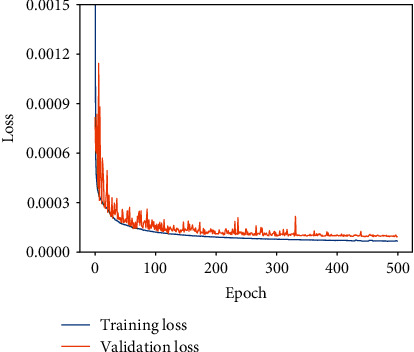
Training and validation losses of the proposed method.

We used the following mean absolute error (MAE) function to optimize (minimize) the MACS-Net error:

(11) $$\begin{array}{c} \text{Loss}\left(G,X\right)=\overset{N}{\underset{i=0}{\sum }}\left|G_{i}-{\hat{X}}_{i}\right|, \end{array}$$

where *N* indicates the number of pixels in the image and *G* and *X* are the ground truth (motion-free) and reconstructed (output) images, respectively. The use of normalized data accelerated the convergence of both the training and validation losses (Figure 6). The regularizing effect afforded by the high connectivity reduced the risk of overfitting during training.

**Figure 7:**
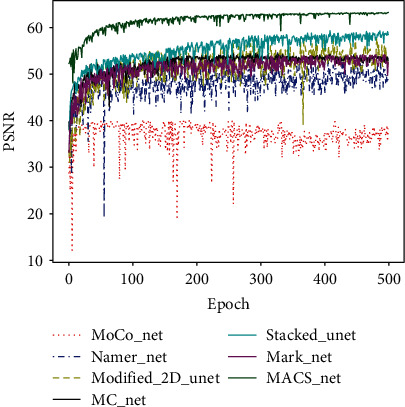
PSNR data acquired during model training.

The PSNR of the validation dataset was assessed during training of the comparative models. When machine learning is supervised, a validation dataset is used to compare the effectiveness of various trained models; this aids the selection of appropriate hyperparameters.  Figure 7 shows that our MACS-Net generates higher and more stable PSNR values than other networks.

## 4. Results and Discussion

The performance of MACS-Net was compared to those of MoCo-Net [11], Modified-2D-Net (which uses a CNN to correct out-of-field-of-view MAs) [12], Namer-Net [26], the MC-Net motion correction network [43], Stacked-Unet (which uses self-assisted priors) [44], and Mark-Net (which performs MA reduction and k-space analysis) [45]. These methods are mainly used in encoder-decoder frameworks, particularly Unet, and executed convolutional operations. Conversely, the proposed MACS-Net method used the Unet framework with “shifted windows” (Swin) transformer block. The Swin transformer block extracted features from the input image and generated a low-dimensional representation, which passes through the Unet to generate the artifacts' free final images. All the comparative methods in this manuscript have been evaluated by the same 256 × 256 size images. The architecture of the neural network being used might be designed to operate efficiently on inputs of a certain size. A 256 × 256 image can contain sufficient information for the model to make accurate predictions or analyses. It is a balance between having enough detail to extract meaningful features and keeping the computational requirements manageable. Processing larger images requires more computational resources, including memory and processing power. Using smaller input sizes [12] can make computations faster and more feasible, especially in real-time applications or when dealing with large datasets, but it may reduce the necessary information of the images. The NRMSE, SSIM, and PSNR values were derived via numerical analyses. The average reconstruction times for both types of motion were computed.  Table 1 lists the similarities and dissimilarities among the methods in terms of the network parameters, up- and down-sampling methods, activation functions, and training time in the same environment. MACS-Net uses patch merging and a new dual upsampling technique instead of the average, Max, or GlobalMax_pooling method; the new technique better represents features. MACS-Net employs the GELU and ELU activation functions instead of the rectified linear unit (ReLU). When learning complicated data patterns, the GELU may be better than the ReLU because the shape of the former is smoother (more continuous). The ELU yields more precise results and converges costs to zero more rapidly than the other functions.

A total of 3,500 images were used to evaluate the performance of all networks.  Table 2 lists the mean and standard deviation NMRSE, SSIM, and PSNR values when motion was brief and moderate; all state-of-the-art techniques were employed. Clinical evaluations of edge sharpness, motion fidelity, and image distortion, as well as diagnostic scores, should be added to these quantitative metrics when evaluating reconstructed images. The post hoc paired *t*-test and one-way analysis of variance (ANOVA) were used to identify statistically significant changes. A *p* value <0.01 indicated statistical significance. All measurements and acceleration parameters differed significantly (*p* < 0.01) according to one-way ANOVA. The observations and numerical analyses show that MACS-Net yielded the best NRMSE, SSIM, and PSNR values. Paired *t*-tests showed that the transformer-based MACS-Net was better than CNN-based methods. Aside from the quantitative results, our new method processes images more rapidly than the other methods. On average, MACS-Net requires 21 s to correct brief MAs, whereas the CNN-based approaches need ≥22 s; the time required in cases with moderate motion was 26 and >26 s, respectively.

**Figure 8:**
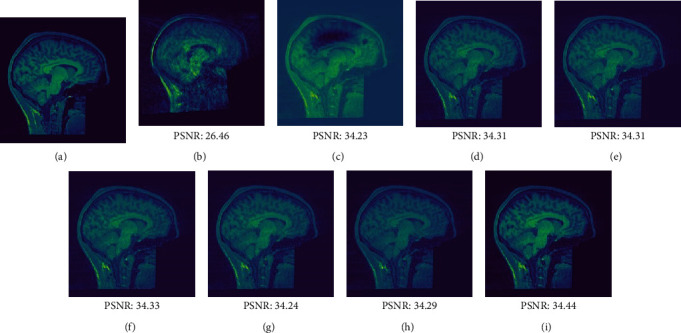
Reconstructed images of slice 82 of the test dataset with brief motion (<5 s). (a) The motion-free ground truth image. (b) The image with an MA. Images corrected by (c) MoCo-Net, (d) Namer-Net, (e) Modified-2D-Net, (f) MC-Net, (g) Stacked-Unet, (h) Mark-Net, and (i) MACS-Net.

**Figure 9:**
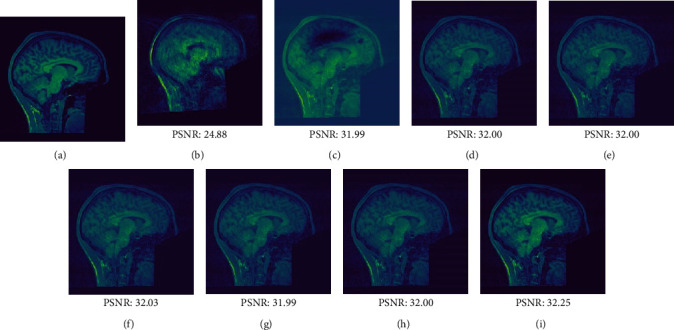
Reconstructed images of slice 82 of the test dataset with moderate brief motion (5–10 s). (a) Motion-free ground truth image. (b) The image with an MA. Images corrected by (c) MoCo-Net, (d) Namer-Net, (e) Modified-2D-Net, (f) MC-Net, (g) Stacked-Unet, (h) Mark-Net, and (i) MACS-Net.

Figures 8 and 9 show the visual assessments (obtained via different methods) of motion-corrected slice 82 images with brief and longer motions, respectively. Both figures include reference images (a), images with MAs (b), and various visual evaluations of the motion-corrected images (c–i). The CNN-based models eliminated MAs effectively, but the reconstructed images were distorted compared to the reference images. MACS-Net precisely corrected the artifacts and reconstructed the images (i) such that they were similar to the reference (a). When motions were brief or moderate, MACS-Net performed better than the CNN-based techniques. Also, MACS-Net outperformed the other approaches in terms of the quantitative values. The PSNR of slice 82 increased from 26.46 to 34.44 dB when motion was brief and from 24.88 to 32.25 dB when motion was moderate. Network generalizability was apparent; MAs in the test dataset were suppressed. MACS-Net optimally removed artifacts because, during image reconstruction, MACS-Net focuses on features essential for accurate diagnosis.

Table 3 summarizes the ablation studies of base Unet [34], Swin Unet [38] with bilinear upsampling, and Swin Unet with PixelShuffle upsampling and proposes Swin Unet with new dual upsampling (combining bilinear and PixelShuffle) approaches for both types of motions. Traditional Unet architectures commonly used transposed convolutions (also known as deconvolutions) for upsampling in the decoder layers. Transposed convolutions learn a set of trainable parameters to upsample feature maps. Swin Unet with bilinear upsampling replaces transposed convolutions with bilinear upsampling during the decoding phase. It provides a smooth interpolation of pixel values compared to transposed convolutions. Swin Unet with PixelShuffle upsampling provides the model with the ability to learn specific rearrangement patterns during training, making it more adaptable to image reconstruction. The dual upsampling approach (Figure 3) has benefited from the efficiency of bilinear upsampling and the ability of PixelShuffle to rearrange feature maps for increased spatial resolution. The proposed method provides the best performance in both types of motions compared to the base Unet and other upsampling modules according to Table 3 outcomes.

In general, conventional methods such as navigator-based respiratory gating, image averaging, and image filtering are widely used in clinical settings to reduce MAs. However, all of these methods have certain limitations; they correct only specific motions or require multiple scans. Recently, DL methods have been used to correct MAs in MRI scans. Typically, an NN is trained on an image dataset with and without MAs, and the trained network is used to remove artifacts from new images. One common approach uses a CNN trained using superresolution imaging; this increases image resolution by adding details lost because of MAs. Another approach employs a GAN to generate images free of MAs by comparing the created and original images. In contrast, the Swin transformer-based model allows a network to learn motion patterns and then filter out MAs. Notably, for both brief (within 5 s) and moderate (within 5 to 10 s) motions, the transformer-based approach outperformed the CNN-based methods, although this method has certain limitations. First, DL models are highly dependent on the quality, diversity, and representativeness of the training data. In this case, only T1-weighted sagittal brain slices have been used. So, it is important to evaluate this model in different clinical settings such as T2-imaging contrast, knee and abdomen datasets, and axial and coronal brain slices. Second, this model is considered a “black box” due to their complex architecture. Therefore, understanding the decision-making process of this model, especially in a medical context, needs further investigation.

## 5. Conclusion

Our MACS-Net architecture uses a new Swin transformer backbone to correct MAs. The new dual upsample module prevents aliasing artifacts. MACS-Net effectively removes MAs from MRI scans and improves image diagnostic quality. Higher image quality often leads to more accurate and reliable diagnostic information. Automated correction of MAs reduces the time required for image analysis and interpretation, allowing for more efficient use of resources. Furthermore, this method can contribute to a more positive patient experience, potentially increasing compliance and reducing anxiety during MRI procedures. However, it is premature to suggest that the Swin transformer can replace convolution; the performance of Swin transformer-based NN architectures must be further evaluated and optimized.

## Figures and Tables

**Table 1:** Comparison of state-of-art motion correction methods.

Year proposed	Method	Parameters	Resizing module	Activation function	Training time (h min)
2018	MoCo-Net	47,120,129	MaxPooling	ReLU	67 20
2019	Namer-Net	893,899	—	ReLU	81 24
2020	Modified-2D-Net	3,363,215	Conv with stride 2	ReLU	23 52
2022	MC-Net	5,496,001	MaxPooling	ReLU, sigmoid	20 44
2022	Stacked-Unet	4,017,966	Average_pooling, GlobalMax_pooling	ReLU	136 49
2023	Mark-Net	1,623,236	MaxPooling	LeakyReLU	23 22
—	Proposed MACS-Net	33,227,907	Patch merging, dual upsampling	GELU, ELU	56 54

**Table 2:** Average NRMSE, SSIM, and PSNR values and reconstruction times of state-of-art techniques analyzing two different motions.

Type	Approach	NRMSE (×10^−2^) Mean ± std	SSIM (×10^−2^) Mean ± std	PSNR (dB) Mean ± std	Time (s)
Brief motion (<5 s)	Zero filled	45.25 ± 0.07	79.43 ± 0.09	18.24 ± 3.05	—
	MoCo-Net	29.06 ± 0.07	88.81 ± 0.07	22.14 ± 3.70	27
	Namer-Net	17.67 ± 0.06	91.28 ± 0.07	26.48 ± 2.47	42
	Modified-2D-Net	17.64 ± 0.06	91.27 ± 0.07	26.49 ± 2.46	22
	MC-Net	17.63 ± 0.06	91.29 ± 0.07	26.50 ± 2.48	27
	Stacked-Unet	17.96 ± 0.06	91.26 ± 0.07	26.34 ± 2.51	29
	Mark-Net	17.70 ± 0.06	91.27 ± 0.07	26.46 ± 2.47	22
	MACS-Net (proposed)	17.51 ± 0.06	91.72 ± 0.07	26.57 ± 2.52	21

Moderate motion (5–10 s)	Zero filled	60.30 ± 0.03	33.86 ± 0.04	15.64 ± 1.54	**—**
	MoCo-Net	31.92 ± 0.07	87.19 ± 0.07	21.25 ± 3.13	38
	Namer-Net	21.26 ± 0.07	89.78 ± 0.06	24.88 ± 2.16	48
	Modified-2D-Net	21.20 ± 0.07	89.78 ± 0.07	24.90 ± 2.15	27
	MC-Net	21.18 ± 0.07	89.82 ± 0.07	24.91 ± 2.17	34
	Stacked-Unet	21.58 ± 0.07	89.75 ± 0.06	24.75 ± 2.20	39
	Mark-Net	21.27 ± 0.07	89.77 ± 0.07	24.88 ± 2.16	33
	MACS-Net (proposed)	21.04 ± 0.07	90.33 ± 0.07	24.99 ± 2.20	26

**Table 3:** Performance of the networks based on different modules.

Motion type	Modules	NRMSE (×10^−2^) Mean ± std	SSIM (×10^−2^) Mean ± std	PSNR (dB) Mean ± std
Brief motion (<5 s)	Base Unet	43.40 ± 0.08	34.46 ± 0.02	16.42 ± 0.76
	Swin Unet with bilinear upsampling	38.96 ± 0.17	79.60 ± 0.04	19.54 ± 1.65
	Swin Unet with PixelShuffle upsampling	31.80 ± 0.13	82.30 ± 0.16	22.48 ± 1.24
	Swin Unet with dual upsampling (MACS-Net)	17.51 ± 0.06	91.72 ± 0.07	26.57 ± 2.52

Moderate motion (5–10 s)	Base Unet	53.10 ± 0.08	34.78 ± 0.01	16.42 ± 0.77
	Swin Unet with bilinear upsampling	49.17 ± 0.03	73.23 ± 0.17	17.24 ± 1.42
	Swin Unet with PixelShuffle upsampling	37.67 ± 0.10	77.60 ± 0.92	21.43 ± 1.15
	Swin Unet with dual upsampling (MACS-Net)	21.04 ± 0.07	90.33 ± 0.07	24.99 ± 2.20

## Data Availability

The dataset is accessible from https://openneuro.org/datasets/ds004173/versions/1.0.2 (last accessed on 15 November 2023).

## Abbreviations

1D: One dimension
2D: Two dimensions
3D: Three dimensions
ANOVA: One-way analysis of variance
CNN: Convolutional neural network
CV: Computer vision
DL: Deep learning
ELU: Exponential linear unit
GAN: Generative adversarial network
GELU: Gaussian error linear unit
IFT: Inverse Fourier transform
MA: Motion artifact
MACS-Net: Motion artifact correction using the Swin network
MC-Net: Motion correction network
MRI: Magnetic resonance imaging
MSA: Multihead self-attention
NAMER: Network accelerated motion estimation and reduction
NN: Neural network
NRMSE: Normalized root mean square error
PSNR: Peak signal-to-noise ratio
ReLU: Rectified linear unit
SLT: Swin transformer layer
SSIM: Structural similarity index measure
STB: Swin transformer block
SW-MSA: Shifted window-based multihead self-attention.
